# Lamins and bone disorders: current understanding and perspectives

**DOI:** 10.18632/oncotarget.25071

**Published:** 2018-04-27

**Authors:** Chiara Gargiuli, Elisa Schena, Elisabetta Mattioli, Marta Columbaro, Maria Rosaria D'Apice, Giuseppe Novelli, Tiziana Greggi, Giovanna Lattanzi

**Affiliations:** ^1^ CNR Institute of Molecular Genetics, Unit of Bologna, Bologna, Italy; ^2^ Rizzoli Orthopaedic Institute, Laboratory of Cell Biology, Bologna, Italy; ^3^ Medical Genetics Unit, Policlinico Tor Vergata University Hospital, Rome, Italy; ^4^ Rizzoli Orthopaedic Institute, Spine Deformity Department, Bologna, Italy

**Keywords:** lamin A/C, LMNA-related congenital muscular dystrophy (L-CMD), hutchinson-gilford progeria syndrome (HGPS), mandibuloacral dysplasia (MADA, MADB), bone turnover

## Abstract

Lamin A/C is a major constituent of the nuclear lamina implicated in a number of genetic diseases, collectively known as laminopathies. The most severe forms of laminopathies feature, among other symptoms, congenital scoliosis, osteoporosis, osteolysis or delayed cranial ossification.

Importantly, specific bone districts are typically affected in laminopathies. Spine is severely affected in LMNA-linked congenital muscular dystrophy. Mandible, terminal phalanges and clavicles undergo osteolytic processes in progeroid laminopathies and Restrictive Dermopathy, a lethal developmental laminopathy. This specificity suggests that lamin A/C regulates fine mechanisms of bone turnover, as supported by data showing that lamin A/C mutations activate non-canonical pathways of osteoclastogenesis, as the one dependent on TGF beta 2.

Here, we review current knowledge on laminopathies affecting bone and LMNA involvement in bone turnover and highlight lamin-dependent mechanisms causing bone disorders. This knowledge can be exploited to identify new therapeutic approaches not only for laminopathies, but also for other rare diseases featuring bone abnormalities.

## INTRODUCTION

Lamins constitute a network of filamentous proteins, the nuclear lamina, underlying the inner nuclear membrane [1]. A type lamins are produced as alternatively spliced products of the *LMNA* gene located on chromosome 1 and include lamin A and C and the minor isoforms lamin C2 and lamin A delta 10 [2]. Prelamin A is the protein precursor of lamin A, which is post-translationally modified at its C-terminal region, where the CaaX motif undergoes farnesylation, carboxymethylation and proteolytic cleavage by ZMPSTE 24 metalloproteinase [2, 3]. The A-type lamins stabilize mechanically the nucleus [4], are involved in chromatin dynamics [5, 6] and stress response [7] and influence several signalling pathways [2, 8–11].

Mutations in *LMNA* gene cause a wide and heterogeneous group of diseases belonging to the category of laminopathies, which may affect skeletal and cardiac muscle, bone, adipose tissue and peripheral nerves and may be associated with accelerated ageing [2, 5]. Bone disorders found in laminopathies are osteoporosis, osteolysis, delayed closure of cranial sutures and scoliosis, the latter being mostly found in congenital muscular dystrophies linked to *LMNA* [2, 5]. Osteoporosis is a common pathology, in which the density and quality of bone are reduced and the risk of fracture is greatly increased. The loss of bone is due to an imbalance between bone resorption and bone production and it seems to be determined by genetic, environmental and inflammatory factors, the latter involved in dysregulation of osteoclast versus osteoblast activity [12]. Osteolysis is a rare disorder associated with prosthesis implantation, tumours and, interestingly, excess exercise of clavicles such as in weight lifters [13]. The process seems to be mediated by inflammatory molecules and possibly caused by repeated stress stimuli [13]. Patent cranial sutures are observed several months after birth in a plethora of genetic bone disorders. It has been suggested that this event is associated with altered bone turnover and hyperactivation of Wnt signaling, which impairs endochondral ossification [14]. Scoliosis is a very common spine defect, which may either occur in severe forms of congenital muscular dystrophy or in other inherited or idiopatic diseases. Laminopathies with bone involvement, including, among others, Hutchinson-Gilford Progeria (HGPS), Mandibuloacral dysplasia type A and B (MADA; MADB) and *LMNA*-related congenital muscular dystrophy (L-CMD) may be considered a genetic model of these bone disorders and further deepening of the role of lamins in bone turnover will provide new tools to identify therapeutic targets for osteoporosis and forms of skeletal dysplasia.

## BONE DISORDERS AND LAMINOPATHIES

### Laminopathies

So far, more than 15 different clinical entities, some of which showing overlapping features, have been defined as laminopathies [5, 13]. Most laminopathies are linked to mutations in *LMNA* gene. Information on *LMNA* gene mutations can be found at http://www.umd.be/LMNA/ [12, 13]. Here we provide a description of the main laminopathies, going in deep into aspects related to bone and skeletal disorders.

### Progeroid laminopathies

Progeroid laminopathies are syndromes characterized by premature aging and include HGPS (OMIM#176670), MADA (OMIM#248370) MADB (OMIM#608612), atypical progeria syndrome (APS, OMIM *150330) and atypical-Werner syndrome (now called Malouf syndrome, OMIM#212112) [2]. Progeroid laminopathies may present partial or generalized loss of subcutaneous fat and skin abnormalities, but all forms share generalized osteoporosis and osteolysis of clavicles, mandible, and phalanges [14–16]. Moreover, dental crowding with malocclusion is usually present in patients affected by progeroid laminopathies [14]. While HGPS and MADB have an early onset, around the first year of life, MADA phenotype becomes evident within the first decade. A severe clinical course characterizes HGPS, with extremely accelerated ageing and cardiovascular impairment leading to death in the first or second decade. Progression of disease is slower in MADB, while an overall milder and slowly progressing phenotype is observed in MADA. Progeroid laminopathies also include RD (OMIM#275210), the most severe disease on the continuum of premature ageing syndromes, with onset before birth and perinatal lethality. However, RD could be also considered a developmental laminopathy, since symptoms arising before birth are typically related to impaired development of skin and growth arrest. Cases of RD have been linked to the c.C1824T *LMNA* mutation, while most RD patients carry the c.1085dupT homozygous mutation in *ZMPSTE24* gene, the gene required for prelamin A maturation. RD was first described in 1985 [42] but only in 2004 Navarro *et al*. [43] identified heterozygous splicing mutations in the *LMNA* gene leading to the production and accumulation of truncated prelamin A or a 1-bp insertion resulting in a premature stop codon in *ZMPSTE24* gene [43, 44]. Other studies from different groups confirmed that, in most of the cases, patients carry *ZMPSTE24* homozygous null mutations [45–47] (Table 1). It seems that a combination of a missense and a nonsense mutation in *ZMPSTE24* gene results in MADB, while two nonsense mutations lead to RD [48]. Typical features of RD include intrauterine growth retardation and fetal hypokinesia deformation sequence. At birth, rigid and tight skin with erosions at flexure sites, epidermal hyperkeratosis and pulmonary hypoplasia are observed.

**Table 1:** List of laminopathies featuring bone phenotype

Disease	Gene	Gene Mutation	Protein Mutation	Inheritance	Bone Phenotype
HGPS	LMNA	c.C1824T	p. Gly608Gly	Heterozygous	Mandible, clavicle and phalanghes osteolysis, delayed closure of cranial sutures, pinched nose, osteoporosis.
MADA	LMNA	c.1580G>A	p.Arg527His	Homozygous^*^	Mandible, clavicle and phalanghes osteolysis, osteoporosis.
MADB	ZMPSTE24	c.1085dupT; c.794A>G	p.Phe361fsX379; p.Asn265Ser	Heterozygous	Mandible, clavicle, phalanghes osteolysis; long bone dysplasia; pinched nose; altered skull calcification; spine and limb osteoporosis.
L-CMD	LMNA	c.745C>T	p.Arg249Trp	Heterozygous	Hyperlordosis, rigid spine, scoliosis.
a-WS	LMNA	c. 584G>C	p. Ala57Pro	Heterozygous	Prominent nasal bones, aracnodactyly, mild scoliosis, osteoporosis
RD	ZMPSTE24	c.1085dupT	Leu 362fsX18	Homozygous	Clavicle osteolysis, small pinched nose, enlarged fontanels.
HHS-S	LMNA	IVS9-12T>G	p.E536fsX14	Heterozygous	Hand and foot short phalanges, clinodactyly, syndactyly, short metatarsal bones.

For each disease, mutated gene, best known gene mutations and amino acid mutation, type of inheritance and bone phenotype are reported. dup, duplication; fs, frame-shift; X, stop. ^*^Most cases.

### Skeletal muscle laminopathies

*LMNA*-related myopathies include different phenotypes such as autosomal dominant Emery-Dreifuss muscular dystrophy (EDMD2, OMIM#181350) [18], autosomal recessive Emery-Dreifuss muscular dystrophy (EDMD3, OMIM#616516), Limb-girdle muscular dystrophy type 1B (LGMD1B, OMIM#159001) and *LMNA*-related congenital muscular (L-CMD, OMIM#613205) [19, 20]. The X-linked form of Emery-Dreifuss muscular dystrophy (EDMD1, OMIM#310300) is due to mutations in *EMD* gene and it is phenotypically similar to EDMD2, although in EDMD2 loss of independent walking is more frequent and the risk for ventricular tachyarrhythmia and dilated cardiomyopathy is higher [21, 24]. Forms of Emery-Dreifuss muscular dystrophy and LGMD1B are clinically characterized by ankle, elbow and spine contractures, muscle wasting and weakness in the scapulo-humero-peroneal districts which may be severe and impair posture and gait [22]. Heart involvement is very frequent, with dilated cardiomyopathy and conduction system defects [23]. The most severe form of muscular laminopathy is L-CMD, a disease with very early onset that impairs ambulation in several cases [20, 25]. Among the small number of affected individuals identified to date, several share the same pathogenic variants c.91G>A, c.745C>T and c.116A>G, suggesting a possible phenotype-genotype correlation [55], although new mutations in *LMNA* gene that lead to L-CMD are being discovered [56, 57]. L-CMD may manifest with different phenotypes that can be classified as 1) severe phenotype with generalized muscular weakness and contractures by birth, 2) ‘dropped head’ phenotype with prominent involvement of axial muscles that generally evolves to rigid spine phenotype and 3) early onset Emery-Dreifuss phenotype. All these conditions generally lead to severe cardiomyopathy, respiratory insufficiency, orthopaedic complications and metabolic disorders in the first decade [25, 59].

Several mechanisms have been proposed for the pathogenesis of muscular laminopathies. In EDMD muscle the transcriptional regulation is defective because of the loss and disorganization of heterochromatin in fibroblast and muscle fibre nuclei [5]. Moreover, nuclear clustering and abnormal expression of proteins linking the nucleus to the cytoskeleton have been demonstrated in muscle cells and mature muscle from patients affected by muscular laminopathies [1, 2]. Importantly, systemic effects have been reported in models of muscular laminopathies, especially those involving the transforming growth factor beta (TGFbeta) axis [3]. This complexity suggests that diverse tissues and organs could be affected by lamin A mutations through diverse pathogenetic pathways still to be elucidated.

### Bone phenotype in laminopathies

**Figure 1:**
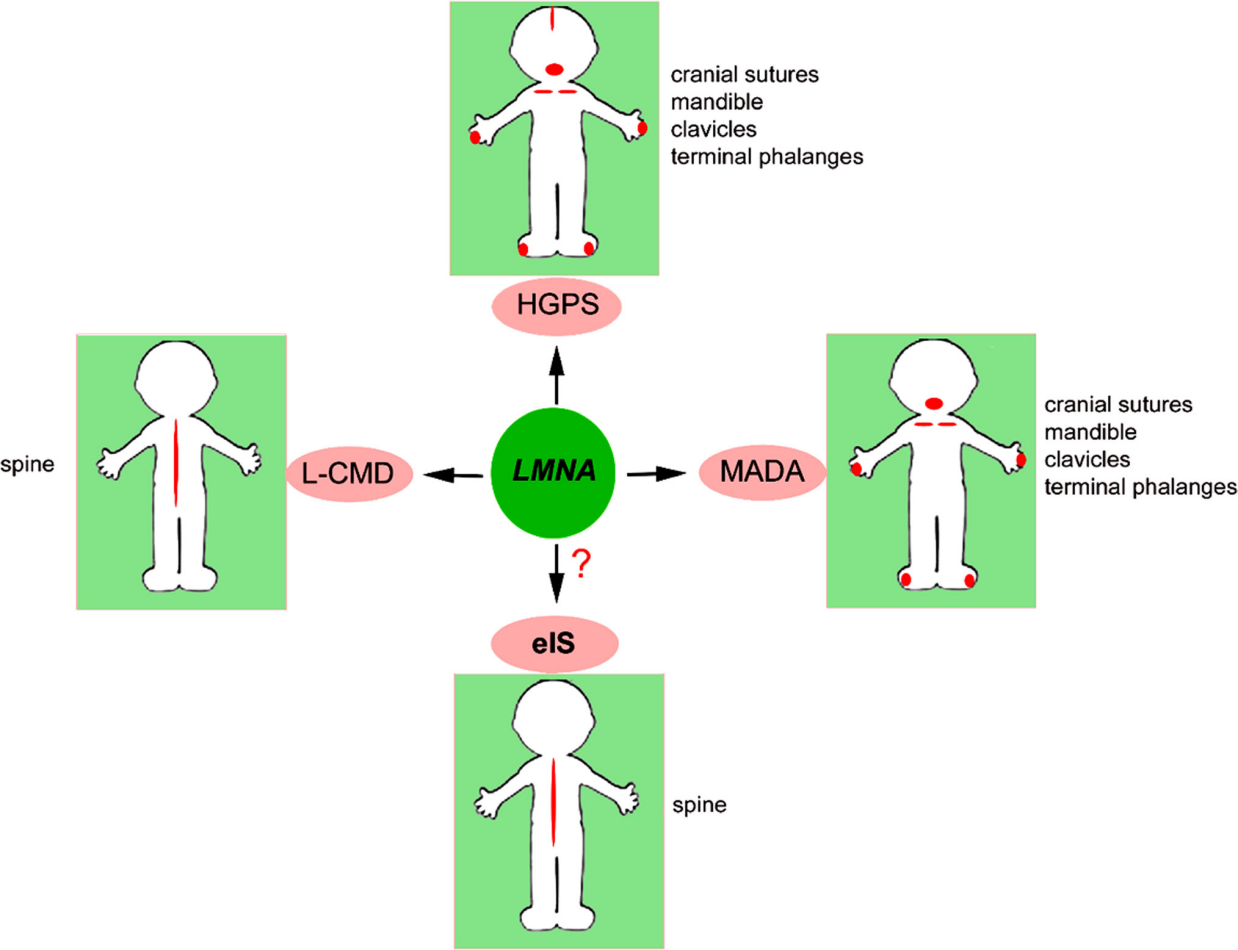
Bone districts targeted by *LMNA* Bone districts affected in *LMNA*-mutated diseases and in eIS are indicated in red and listed next to each picture.

Bone is affected in several laminopathies, as schematically depicted in Figure 1.

Two hot spots in *LMNA* gene are linked to syndromes featuring bone disorders, the c.C1824T mutation (p.G608G) associated with HGPS and a few cases of RD, and the c.1580G>A mutation (p.R527H), linked to MADA. Moreover, the c.745C>T (p.R249W) *LMNA* mutation is associated with most cases of L-CMD (http://www.umd.be/LMNA/).

HGPS features midface hypoplasia, micrognathia, delayed closure of the cranial suture and generalized osteodysplasia with osteolysis and pathologic fractures [26]. The bone phenotype of HGPS and other laminopathies with skeletal involvement is detailed in Table 1. Children affected by HGPS typically appear normal at birth, but the characteristic features appear within a year and progressively worsen [26].

MADA is also characterized by postnatal growth retardation, craniofacial anomalies associated with mandible hypoplasia, osteolysis of terminal falanges and clavicles, delayed closure of the cranial suture [17, 27, 28, 29–32] (Table 1). In MADA, skeletal disorders usually appear in the first decade. However, despite the slow and benign clinical course of the whole disease, progress more rapidly than in HGPS [17, 28, 29, 33].

So far, less than ten MADB patients who carry mutations in *ZMPSTE24* gene have been described in the literature. In all the cases, the phenotype manifested is more severe compared to MADA patients, although target organs and clinical features are super imposable [34–38]. An important example has been described by Cunningham *et al*. [17]. The patient studied carried two heterozygous compound mutations in the *ZMPSTE24* gene and showed unusual skeletal features in addition to the others including premature tooth eruption, beaking of the vertebrae, the development of amorphous subcutaneous calcific deposits, progressive submetaphyseal changes at the proximal ends of long bones and severe osteoporosis with fractures and delayed healing. The skull showed typical features of MADA with mandibular hypoplasia, dental crowding and failure of fusion of the anterior mandibular rami [17]. At birth the unusual presence of two erupted teeth was noticed together with the failure of ossification of the occipital bone [39]. At the age of 8 months the patient displayed bone resorption in the terminal phalanges and clavicles. At the age of 4 years, radiographs showed that neither clavicle was visible but amorphous calcific masses were present in their place, which persisted into adolescence [17]. There was marked anterior “beaking” of the lower thoracic and upper lumbar vertebrae. It has been observed symmetrical erosions of the proximal submetaphyseal regions of long bones with the formation of amorphous calcific masses in the humeri, tibiae and femora at the age of 3 years and the radii at the age of 6 years [17]. Moreover, it has been noticed that these changes were progressive and when the patient reported bone fractures, these were very slow to heal. Another interesting MADB case has been described by Ben Yaou *et al*. [40]. The patient had a very early onset of bone disorders consisting of decreased spine and limb bone density, multiple spontaneous fractures, osteolysis of femoral head and cranial sutures still open at 15 years of age [40]. These findings together suggest that patients with MAD due to *ZMPSTE24* mutations have a more severe skeletal phenotype than those with *LMNA* mutations [17, 38, 40] (Table 1).

A bone phenotype resembling MADA, MADB and HGPS features is observed in RD, although the onset of osteolytic processes is at birth and progression is impressive, with almost complete resorption of clavicles a few days or weeks after birth [41] (Table 1). Bone mineralization defects, thin dysplastic clavicles, kyphoscoliosis, joint contractures, widened sutures and generalized arthrogryposis are present in RD [48].

For diagnostic purposes, although a typical pathological pattern of laminopathic bone has not been univocally recognised, appearance of mandibular hypoplasia and clavicle osteolysis can be considered as an indication of *LMNA*-linked bone disease with progeroid features.

Bone involvement is also observed in Slovenian-type Heart Hand syndrome (HHS-S, OMIM#610140), an extremely rare disease caused by a mutation in intron 9 of *LMNA* (c.1609-12T>G or IVS9-12T>G) affecting the splicing mechanisms and producing a truncated protein lacking the carboxy-terminal domain [50]. Heart hand syndromes are a group of heterogenous disorders that also comprise Holt-Oram syndrome (OMIM#142900), heart-hand syndrome type 2, heart-hand syndrome type 3. Altered bone development at the hands and malformations of the upper limbs and brachydactyly with mild hand involvement and severe foot involvement are typical of HHS-S [49, 50] (Table 1). HHS-S is also characterised by proximal skeletal muscle weakness and atrophy, joint contractures, sinoatrial and atrioventricular conduction disease, sudden death due to ventricular tachyarrhythmia and dilated cardiomyopathy [50].

In L-CMD, scoliosis is often associated with muscular dystrophy [51–53] and may manifest as hyperlordosis [54]. L-CMD may present with a severe picture in the first six months of life (absence of head or trunk support) or with progressive loss of head support after acquisition of sitting or walking ability (dropped head syndrome). Often hypotonia and weakness of the axial-cervical muscles is rapidly progressive, followed by more slowly progressive weakness of the proximal upper limbs and distal lower limbs. With time, the characteristic findings are head lag, thoracic and lumbar spinal hyperextension (rigidity), lower limb contractures, and talipes equinovarus but no significant upper limb contractures [58].

## LAMINS AND SCOLIOSIS

### Scoliosis

Scoliosis is an alteration of the normal morphology of the spinal column with a lateral curvature or deviation of the spine greater than 10°, associated with vertebral rotation which makes scoliosis a three-dimensional deformity. The clinical aspects of this disease include muscolo-skeletal deformities, back pain and vertebral defects such as hemi-vertebrae, rigid spine, butterfly and wedged vertebra, unsegmented bars. As the spinal curvature progresses and if the deformity occurs in the thoracic region, pulmonary function may be compromised. Restrictive lung function is associated with spinal curvature that approaches 90° [60]. Four types of scoliosis have been described: congenital, idiopathic, neuromuscular and syndromic.

Early onset scoliosis refers to spine deformity that is present before 10 years of age. The idiopathic scoliosis is determined by mostly unknown causes [61] and appears to be a multifactorial disease with a contribution of genetic, biochemical, neurological and muscular defects [62]. Several models for the inheritance of idiopathic scoliosis have been proposed but nowadays none of them have been validated. Dominant forms with variable penetrance, autosomal recessive, multifactorial, X-linked and polygenic models have been reported [63–67]. Segregation analysis pointed out a single gene as a major determinant of idiopathic scoliosis [68]. Different candidates, including *COL1A1*, *COL1A2*, *COL2A1*, *FBN1* and elastin genes have been examined by linkage studies with no results [69, 70]. An original study performed on a sample of parent-offspring, pointed out a linkage and an association between an allele marker on *MATN1* gene, that maps on locus 1p35, and idiopathic scoliosis [71]. Among neuromuscular disorders, Duchenne and Becker muscular dystrophy and diverse forms of congenital muscular dystrophy are associated with severe scoliosis as disease progresses [72].

### Lamins and scoliosis

L-CMD typically features contractures of the spine, hips, knees and Achilles tendons, together with pronounced lumbar hyperlordosis, scoliosis and rigid spine [54]. The origin of contractures in L-CMD is not obvious. It might involve tendon fibrosis, asymmetric muscle deterioration or primary skeletal defects. All these hypothesis are currently under investigation. Our group has identified an effect of laminopathic secretome on tenocyte fibrosis, which could well contribute to the onset of contractures and bone deformities (Bernasconi *et al.*, in preparation).

In a pilot study, we tested the hypothesis that *LMNA* mutations could also occur in early onset idiopathic scoliosis. *LMNA* gene was analysed by Sanger sequencing in blood samples from 12 patients affected by early onset idiopathic scoliosis, but pathogenetic variants were not found in any of the examined samples. Differently from what observed in HGPS and MADA nuclei, where nuclear shape abnormalities, increase of prelamin A levels and chromatin disorganization were detected, neither lamin A/C localization, prelamin A processing or protein expression levels nor nuclear shape were altered in cells from idiopathic scoliosis (Gargiuli *et al*., in preparation). A panel of nuclear envelope genes as well as a panel of genes involved in other bone disorders are currently under investigation in these samples.

## LAMINS AND BONE TURNOVER

### Bone turnover

The maintenance of an adequate bone mass depends on the controlled and timely removal of old, damaged bone. Osteoblasts and osteoclasts interact at the bone surface to balance bone formation and resorption and maintain bone homeostasis during skeletal growth in childhood, skeletal remodelling in adolescence, repair after fracture or microfracture and in response to local biomechanical influences. Osteoclasts are the only bone resorptive cells. They arise by cytokine-driven proliferation and differentiation of monocyte precursors that circulate within the hematopoietic cell pool [73, 74]. Osteoclasts have the function to resorb bone through secretion of hydrochloric acid and proteases in order to dissolve the calcified bone matrix. Acidification of the resorption compartment is reached through the activity of osteoclast specific V-ATPase, which provides an active proton transport [75]. Chloride, on the other hand, is secreted by the CIC-7 antiporter [76].

The increase of osteoclast activity is observed in many pathologies characterized by bone loss, such as osteoporosis, rheumatoid arthritis, bone metastasis and in paediatric diseases such as phenylketonuria and 21-hydroxylase deficiency [77].

Bone marrow stromal cells provide physical support for nascent osteoclasts and produce soluble and membrane-associated factors that are essential for the proliferation and differentiation of osteoblast precursors [77]. The cytokines required for osteoclast formation are RANK ligand (RANKL) and M-CSF. These factors are produced primarily by bone marrow stromal cells, osteoblasts and activated T cells [78]. Osteoclast formation is driven by contact with bone mesenchymal cells, which express RANKL [79]. RANKL is a member of the tumour necrosis factor (TNF) superfamily, which exists as membrane-bound protein or as a soluble form (sRANKL) obtained by metalloproteinase cleavage [80, 81]. RANKL binds to the transmembrane receptor RANK expressed on the surface of osteoclasts and osteoclast precursors. RANKL also binds to osteoprotegerin (OPG), a soluble decoy receptor produced by numerous hematopoietic cells. Thus, OPG, by sequestering RANKL and preventing its binding to RANK, functions as a potent anti-osteoclastogenic cytokine [82].

RANKL promotes the differentiation of osteoclast precursors from an early stage of maturation into fully mature multinucleated osteoclasts. RANKL is also capable of activating mature osteoclasts, thus stimulating the capacity of these cells to resorb bone. M-CSF induces the proliferation of early osteoclast precursors, the differentiation of more mature osteoclasts, the fusion of mononucleated pre-osteoclasts and increases the survival of mature osteoclasts. Other factors have been described to affect osteoclastogenesis, such as parathyroid hormone, parathyroid hormone-related protein, glucocorticoids, interleukin (IL)-1, IL-6, IL-7, IL- 11, TNF-α, prostaglandin-E_2_ and TGFbeta 2 [83–86]. Many of these factors exert most of their osteoclastogenic activity by inducing RANKL expression in osteoblasts [87].

New bone formation involves ossification, a well-orchestrated, complex process in which crystals of calcium phosphate are produced by osteoblasts and deposited within the bone's fibrous matrix [88].

Osteoblasts are derived from MSC progenitors, which reside in the bone marrow close to haematopoietic stem cell (HSC) niches. MSCs differentiate into preosteoblasts and then mature osteoblasts [89]. Their own location enables MSCs to maintain bone marrow homeostasis and to regulate the maturation of both haematopoietic and non-haematopoietic cells. Growth factors such as TGFbeta, released from the bone matrix during the resorption process, participate in regulation of osteoblast differentiation and function [90].

Recent evidence has linked altered processing of lamin A or lamin A/C deficiency to cellular features of osteoporosis [83, 91–94], suggesting that lamin A/C could play an important role in the regulation of bone turnover.

### Bone disorders in animal models of laminopathies

Several animal models have been generated to better understand laminopathic phenotypes associated with osteolysis and osteoporosis [95]. These phenotypes always manifest with accelerated ageing, as in HGPS or MADA [2]. *Zmpste24*^–/–^ mice were the first developed animal model of lamin-linked accelerated ageing [96, 97]. These animals accumulate toxic levels of wild-type prelamin A due to lack of the processing endoprotease Zmpste24. The bone phenotype of *Zmpste24*^–/–^ mice consists of kyphosis, cranial and teeth malformation [96, 98]. Moreover, *Zmpste24^-/-^* mice show bone fractures in multiple locations aside from the ribs, including the scapula, clavicle, sternum, zygomatic arch, mandible, and humerus. The bone abnormalities in *Zmpste24*^–/–^ mice do not seem to be associated with increased bone turnover [97]. Also, bones from *Zmpste24*^–/–^ and *Zmpste24*^+/+^ mice contain similar numbers of osteoclasts, as judged by staining for tartrate-resistant acid phosphatase [97].

HGPS clinical features are recapitulated in p.G608G *LMNA* mice expressing human mutated prelamin A (progerin). These mice display severe bone abnormalities, including spontaneous bone fractures in the extremities, poorly mineralized bones and defects in dentition [99].

Yang *et al*. in 2006 [100] generated a mutant mouse model, the *Lmna*^HG/HG^, expressing progerin in the homozygous state. These mice show severe bone abnormalities and bone fractures at the extremities, associated with complete absence of adipose tissue. Moreover, heterozygous *Lmna^HG/+^* mice appear normal for the first 3 weeks of life. By 6–8 weeks, however, both male and female *Lmna^HG/+^* mice begin to lose weight, display significantly less subcutaneous and abdominal fat, show kyphosis and osteolytic lesions in the ribs, predisposing to rib fractures near the costovertebral junction [100].

Finally, progeroid mice that accumulate progerin due to expression of the p.G609G *Lmna* mutant (corresponding to the human G608G *LMNA* mutation) exhibit growth retardation, abnormal gait, immobility of the joints, deformations of the skeleton and changes in bone mineral density [101]. The authors performed a microcomputed tomography analysis of tibias, skull, and vertebral column revealing profound bone alterations in *Lmna*^G609G/G609G^ mice compared to wild-type mice. Thus, the tibias of mutant mice show a reduction in bone density and cortical thickness as well as an increased porosity. Skulls show a clear size reduction and smaller lower incisors, whereas vertebral column analysis points out a marked lordo-kyphosis in mutant mice.

Another mouse model has been generated by the group of Eriksson [31]. This model features inducible and tissue-specific expression of the most common HGPS mutation, p.G608G *LMNA*, in osteoblasts and odontoblasts. At the age of 5 weeks, HGPS mutant mice show growth retardation, imbalanced gait and spontaneous fractures. Histopathological examination points out an irregular bone structure, characterized by widespread loss of osteocytes, defects in mineralization and a hypocellular red bone marrow. Computed tomography analysis demonstrated impaired skeletal geometry and altered bone structure. The skeletal defects, which resembled the clinical features reported for bone disease in HGPS patients, are associated with an abnormal osteoblast differentiation. From the molecular point of view, the osteoblast-specific expression of the HGPS mutation increases DNA damage and affects Wnt signalling [97]. In the teeth, irregular dentin formation, as previously demonstrated in human progeria cases, causes severe dental abnormalities affecting the incisors. The observed phenotype also shows similarities to reported bone abnormalities in aging. All together, these models may help to uncover bone defects associated with aging and pathogenetic mechanisms of rare and common diseases featuring bone loss and abnormal bone turnover.

### Lamin role in bone turnover

**Figure 2:**
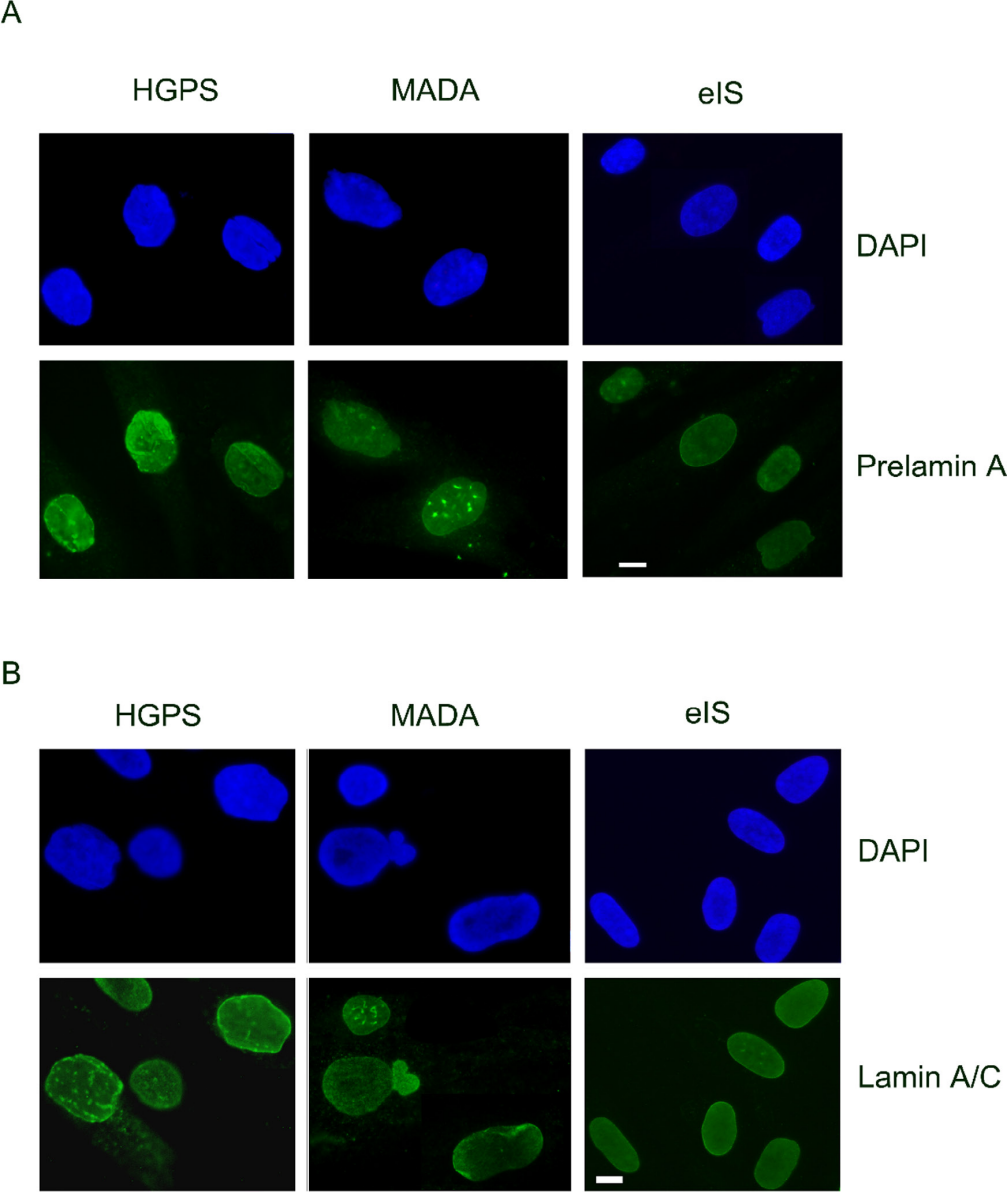
Prelamin A processing, lamin A/C levels and nuclear morphology are not affected in early onset idiopathic scoliosis (eIS) (**A**) DAPI and prelamin A (Santa Cruz Sc-6214 antibody) staining in HGPS, MADA and eIS fibroblasts. (**B**) Lamin A/C (Santa Cruz Sc-6215 antibody) staining in HGPS, MADA and eIS fibroblasts. DAPI has been used to counterstain nuclei. Representative images of three different HGPS, MADA and eIS fibroblast cultures are shown. Images were taken using a Nikon Eclipse Ni-U fluorescence microscope by the NIS AR software.

With increasing age, adipogenic differentiation of MSCs increases and the amount of bone forming cells decreases, which ultimately leads to bone loss [102]. However, the factors that stimulate the age-related shift in MSC differentiation from osteogenesis to adipogenesis are yet to be fully elucidated. A main feature of laminopathies with bone disorders is accumulation of toxic levels of prelamin A in cells. This is observed in MADB, HGPS and MADA nuclei, where nuclear shape abnormalities, increase of prelamin A at the nuclear rim and chromatin disorganization occur (Figure 2).

To obtain a suitable cell model to study prelamin A effects on osteoclasts, our group used two prelamin A processing inhibitors, FTI-277 or AFCMe, to accumulate non-farnesylated or farnesylated prelamin A respectively, in peripheral blood monocytes induced to differentiate towards the osteoclastic lineage [91]. We demonstrated that monocytes subjected to FTI-277 treatment and mostly those subjected to AFCMe administration, differentiate towards the osteoclastic lineage more efficiently than untreated monocytes, in terms of number of multinucleated giant cells and mRNA expression of osteoclast-related genes [91]. On the other hand, the bone resorption activity of osteoclasts obtained in the presence of high prelamin A levels is lower with respect to control osteoclasts. In addition, we showed that there is an augmented secretion of cathepsin K [91].

In 2011, our group analysed, for the first time, an osteoblast primary culture derived from the cervical vertebrae of a MADA patient bearing the homozygous p.R527H *LMNA* mutation. MADA osteoblasts showed nuclear abnormalities typical of laminopathic cells, but they proliferated in culture and underwent differentiation upon stimulation [92]. Osteoblasts showed good differentiation activity with proper production of bone mineral matrix [92]. These findings are also supported by a study performed by the Misteli's group in MSCs, showing normal differentiation of laminopathic MSCs towards the bone lineage [104]. In 2015, the group of Duque [105] published interesting results on the involvement of *LMNA* mutations in osteogenesis. They showed that MSCs with high level of *LMNA* expression exhibit higher rate of osteogenesis associated with high β-catenin activity [105]. Overexpression of *LMNA* in MSCs resulted also in lower level of adipogenesis. This experiment points out that expression of lamin A/C shifts the MSCs commitment from adipogenesis to osteogenesis [105], in agreement with their previous data showing that downregulation of *LMNA* by siRNA impairs osteogenic differentiation [93].

Our published data also showed higher osteoclast differentiation and matrix digestion rate in the presence of MADA osteoblast medium with respect to normal osteoblast medium [92]. This was due to abnormal cytokine secretion determined by mutated lamin A, as later confirmed in an experimental model [83]. In fact, TGFbeta 2 and OPG expression were enhanced in MADA osteoblasts while the RANKL/OPG ratio was diminished and TGFbeta 2 secretion was increased [92]. Importantly, TGFbeta 2 triggered osteoclast differentiation and activity, while inhibition of TGFbeta 2 by a neutralizing antibody abolished the effect of MADA conditioned medium on osteoclast differentiation [92]. These data demonstrate an altered bone turnover in MADA, caused by upregulation of bone-derived stimulatory cytokines, which activate non-canonical osteoclast differentiation.

Moreover, a study performed by our group [83] in 2015 on osteoblast-like U2-OS cells demonstrated that wild-type lamin A downregulates TGFbeta 2 levels. On the other hand, a mutated form of prelamin A associated with MADA, as well as a farnesylated form associated with another progeroid laminopathy [103], fail to downregulate TGFbeta 2 causing increase of secreted TGFbeta 2 which, in turn, activates the AKT/mTOR pathway [4]. The mutated form of prelamin A found in MADA also elicits upregulation of cathepsin K and OPG levels [4].

As detailed in the previous paragraph, altered bone turnover associated with osteoporosis has been described in mouse models of progeroid laminopathies [106]. In these mouse models, zoledronate has been proven to rescue bone mineral density [106]. Based on this observation, and mostly on the evidence that zoledronate avoids accumulation of farnesylated forms of prelamin A, several groups have attempted a therapy in HGPS, MADB and MADA patients based on the use of zoledronate in combination with farnesyltransferase inhibitors and/or statins [106–109]. The results of one of these trials have in fact demonstrated beneficial effects of zoledronate on bone mineral density in patients, although undesired side effects on the cardiovascular system have been also reported [110]. However, since osteolysis appears to be a generalized feature of all MAD forms [111], anti-resorptive therapy may be considered an option to prevent or delay irreversible bone deformities [112].

## CONCLUSIONS

**Figure 3:**
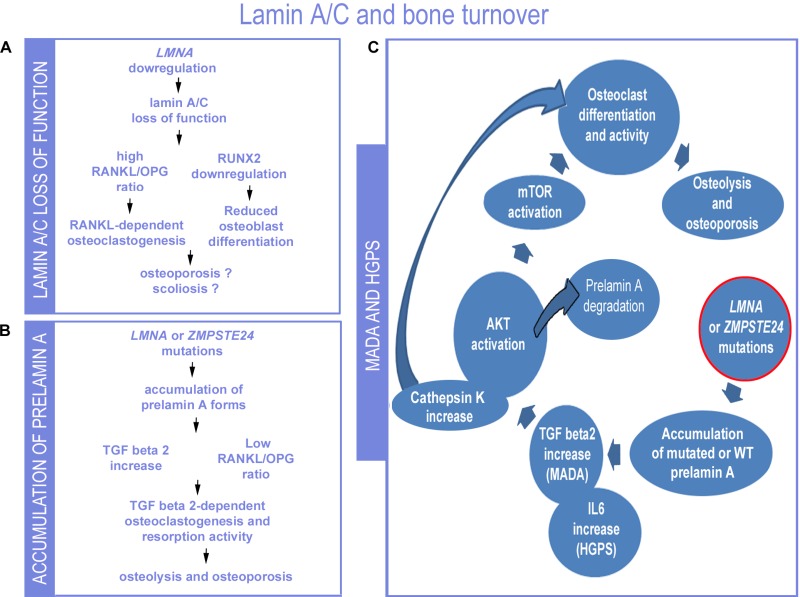
Proposed lamin-dependent pathogenetic mechanisms of bone disorders (**A**) The condition of *LMNA* downregulation or loss of lamin A/C function as described in Lmna null mouse cells is summarized [93, 116]. (**B**) The condition determined by *LMNA* or *ZMPSTE24* mutations that cause prelamin A accumulation is depicted (this paper). (**C**) Data so far obtained in MADA or HGPS studies are summarized in the scheme [83, 91, 92, 101]. While low levels of lamin A/C or loss of lamin A/C expression determines reduced number of osteoblasts, prelamin A or progerin accumulation does not affect or positively affect osteoblast formation and induces osteoclastogenesis and bone resorption. In MADA cells, elevated TGFbeta 2 levels are due to loss of negative regulation of TGFbeta 2 expression associated with *LMNA* mutations. Downstream of TGFbeta2, AKT-mTOR activity and osteoclastogenesis are observed, along with increased secretion of cathepsin K, also linked to bone resorption. Activation of AKT has been linked to prelamin A degradation. IL6 increase has been determined in progeroid mice carrying the G609G *Lmna* mutation and induces osteoclastogenesis.

The involvement of lamin A/C and prelamin A in mechanisms regulating bone turnover is now obvious (Figure 3). Lamin A/C affects both osteoblast and osteoclast differentiation and resorption activity. In particular, *LMNA* mutations associated with progeroid syndromes and causing accumulation of prelamin A forms (Figure 3) increase the rate of osteoclastogenesis and osteolytic activity, while favouring osteoblast differentiation [92]. Conversely, absence of lamin A/C expression, negatively impacts on osteoblast differentiation as well as on osteoclastogenesis (Figure 3) [113]. Of note, absence of *LMNA* expression causes remodelling of the nuclear envelope through upregulation of the nuclear membrane protein MAN1 [113], a finding that involves the nuclear envelope as a whole in bone turnover. In this respect, it is noteworthy that mutations in *MAN1* (also known as *LEMD3*) gene cause osteopoikilosis, a disease characterized by increased bone density [114, 115].

On the other hand, loss of lamin A/C functionality may impact on skeletal development and in particular may contribute to the onset of contractures and scoliosis through mechanisms involving tendon fibrosis, muscle wasting and possibly secretome-dependent effects on bone mineral density [83, 113].

### Consent for publication

Informed consent of the patient has not been provided because the data were analysed anonymously.

### Availability of data and materials

Data sharing not applicable to this article as no datasets were generated or analysed during the current study.

### Ethics approval

The biobank BioLaM for scientific research that provided the cell cultures has been approved by the “IOR Ethics Committee” on 05/09/2016.Prot. gen 0018250 - 01-13 “.

## Abbreviations

APS: Atypical Progeria Syndrome
L-CMD: LMNA-related Congenital Muscular Dystrophy
HGPS: Hutchinson-Gilford Progeria Syndrome
MADA: MADBMandibuloacral Dysplasia type A or type B
eIS: Early onset idiopathic scoliosis
EDMD2: Dominant Emery-Dreifuss muscular dystrophy
LGMD1B: Limb-girdle muscular dystrophy type 1B
EDMD1: X-linked form of Emery-Dreifuss dystrophy
RD: Restrictive Dermopathy
HHS: Heart Hand Syndrome
ZMPSTE24: zinc metallopeptidase STE24
TGFbeta 2: Transforming growth factor beta 2
RANK-ligand RANKL: Receptor Activator of Nuclear Factor κ B
V-ATPase: Vacuolar-type H+-ATPase
CIC-7: Cl-/H+ antiporter
M-CSF: macrophage colony-stimulating factor
FTI-277: farnesyl transferase inhibitor
AFCMe: N-Acetyl-S-farnesyl-L-cysteine-methylester
AKT: serine/threonine kinase 1
mTOR: mechanistic target of rapamycin
LEMD3/MAN1LEM: domain containing protein 3
OPG: osteoprotegerin
IL: interleukin
HSCs: haematopoietic stem cells
MSCs: mesenchymal stem cells.
